# Metabolic dysfunction-associated steatotic liver disease attenuates the predictive value of the triglyceride–glucose index for carotid plaque: evidence of insulin resistance-independent pathways

**DOI:** 10.3389/fendo.2025.1696652

**Published:** 2025-10-16

**Authors:** Fang Zhang, Zheng Wang, Han Zhang, Yu-Qiang Zuo

**Affiliations:** ^1^ Department of Endocrine, the Second Hospital of Hebei Medical University, Shijiazhuang, Hebei, China; ^2^ Department of Respiratory, the Second Hospital of Hebei Medical University, Shijiazhuang, Hebei, China; ^3^ Department of Physical Examination Center, The Second Hospital of Hebei Medical University, Shijiazhuang, Hebei, China

**Keywords:** TyG index, insulin resistance, MASLD, carotid plaque, effect modification

## Abstract

**Background:**

Insulin resistance (IR), a key driver of atherosclerosis, is associated with cardiovascular risks such as carotid plaque formation. While the triglyceride–glucose (TyG) index has been established as a reliable surrogate marker of IR, its association with carotid plaque formation in individuals with metabolic dysfunction-associated steatotic liver disease (MASLD) remains to be explored.

**Purpose:**

The study investigated whether MASLD modified the association between the TyG index and carotid plaque, hypothesizing that MASLD-specific metabolic pathways attenuate the association between the TyG index and carotid plaque.

**Methods:**

A cross-sectional study was conducted in 3414 participants who were stratified into MASLD (n=796) and non-MASLD (n=2618) groups. MASLD was diagnosed by ultrasonography. The TyG index was calculated and its correlation with carotid plaque was evaluated by multivariable logistic regression adjusting for age, sex, body mass index, waist circumference, and metabolic factors. The interaction effects between the TyG index and MASLD status on carotid plaque prediction were evaluated. Receiver operating characteristic analysis compared the discriminatory ability of the TyG index across subgroups.

**Results:**

In the total cohort, a higher TyG index showed a borderline non-significant association with carotid plaque after comprehensive adjustment for covariates including waist circumference (OR: 1.254, 95% CI: 0.985–1.596; *P*=0.066). However, stratification by MASLD status revealed a critical effect modification. The TyG index remained a strong, significant predictor in the non-MASLD subgroup (OR: 1.436, 95% CI: 1.066–1.935; *P*=0.017) but was not associated with plaque in the MASLD subgroup (OR: 0.746, 95% CI: 0.453–1.228; *P*=0.249). This modification was confirmed by a significant TyG index–MASLD interaction term (OR: 0.532, 95% CI: 0.303–0.937; *P*=0.029). ROC analysis confirmed the markedly lower discriminatory power of the TyG index in participants with MASLD (AUC: 0.523 vs. 0.629 in non-MASLD, *P* < 0.001).

**Conclusion:**

MASLD weakened the association between the TyG index and carotid plaque, suggesting that the underlying pathophysiology may involve mechanisms that alter or decouple the relationship between IR and atherosclerosis.

## Introduction

Cardiovascular diseases are the leading cause of mortality globally (1). Insulin resistance (IR) is the fundamental driver of atherosclerosis pathogenesis (2). The triglyceride–glucose (TyG) index is a reliable, robust, accessible surrogate marker, and is an alternative to IR assessment, demonstrating strong associations with subclinical atherosclerosis and cardiovascular disease (3). Recent studies that evaluated the correlation between IR and subclinical atherosclerosis showed that only the TyG index correlated significantly with subclinical atherosclerosis, especially the carotid artery intima-media thickness (4). However, the role of the TyG index in populations with metabolic dysfunction-associated steatotic liver disease (MASLD), a condition also closely linked to IR, remains unexplored.

MASLD affects over 30% of adults globally and is an important and independent risk factor for atherosclerosis development (5, 6). While IR contributes to both MASLD and atherosclerosis, MASLD may independently accelerate atherosclerosis by hepatic pathways such as coagulation abnormalities, the sympathetic nervous system, and dysbiosis of the gut microbiota) (7–10). This raises the hypothesis that MASLD-specific mechanisms could decrease the predictive value of the TyG index for carotid plaque.

Prior research demonstrated an association between the TyG index and cardiovascular disease and stroke (11, 12), but none have investigated its interaction with MASLD. Our study bridged that gap by analyzing whether MASLD modified the TyG index–carotid plaque relationship. The findings could improve risk stratification, particularly in high-MASLD populations.

## Methods

### Study design and population

**Figure 1:**
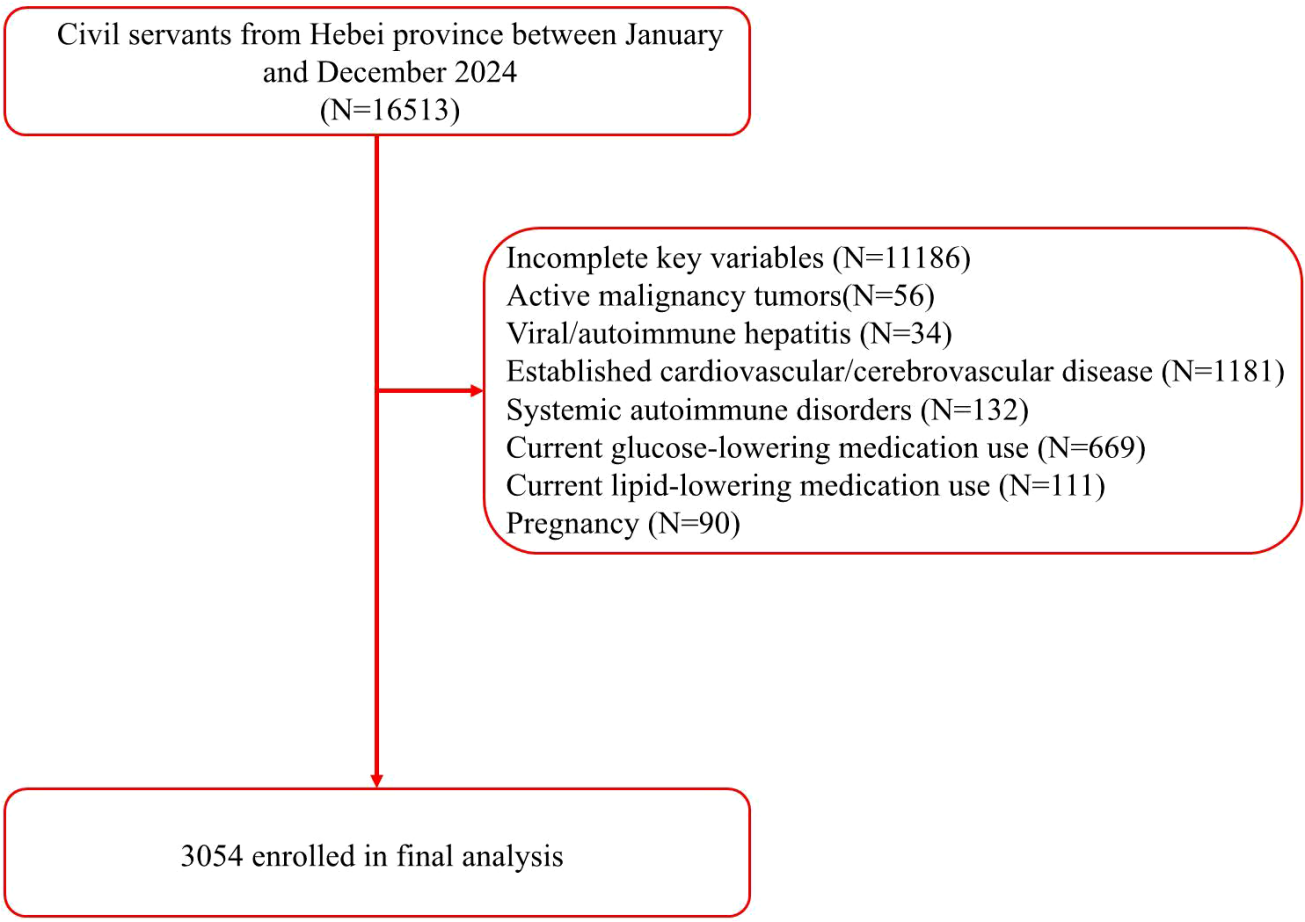
Participant enrollment flowchart.

This cross-sectional study analyzed retrospective data from adults (≥18 years of age) undergoing routine annual health check-ups at the physical examination center of the Second Hospital of Hebei Medical University between January and December 2024. Participants were excluded if they had hepatitis, autoimmune liver disease, established cardiovascular disease, pregnancy, current use of lipid-lowering or glucose-lowering medications, prior carotid surgery, or missing key variables. Notably, 780 participants were excluded due to the use of lipid-lowering or glucose-lowering medications, among whom 442 (56.67%) had MASLD (**Figure 1**). The participant characteristics included demographic data, and concurrent fasting blood tests, carotid ultrasound, and liver ultrasound. The study was approved by the institutional ethics committee of the Second Hospital of Hebei Medical University (no. 2022-R341). Due to the retrospective nature of the study, written informed consent was waived. All personal identifiers were removed to ensure full anonymization, and data handling followed strict confidentiality protocols. The study complied with national and international ethical guidelines for research including human subjects. The sample size was estimated based on prior studies reporting the TyG index–carotid plaque associations (13, 14). Assuming an odds ratio (OR) of 1.4 for the TyG index–carotid plaque association in non-MASLD populations (α=0.05, power=90%), 796 MASLD and 2618 non-MASLD participants provided > 95% power to detect a significant interaction effect (OR <0.60) between the TyG index and MASLD status.

### Data collection and measurement

Baseline demographic and clinical data included sex, age, height, weight, pulse rate, systolic blood pressure (SBP), diastolic blood pressure (DBP), and waist circumference (WC). Blood pressure and pulse measurements were obtained by trained nurses using automated sphygmomanometers (Omron HEM-7136) following standard protocols. Participants remained seated in a quiet environment for at least 5 minutes before measurements were taken. Blood samples were collected and analyzed after fasting for 8–12 hours. The serum was separated by centrifugation (3000 rpm, 10 min) within 30 minutes of collection. Samples were either analyzed immediately or stored at −80 °C until they were assayed. Serum triglycerides (TG), total cholesterol (TC), high-density lipoprotein cholesterol (HDL-C), low-density lipoprotein cholesterol (LDL-C), and fasting plasma glucose (FPG) were measured using enzymatic methods (Beckman Coulter AU5800). For index calculations, TG and FPG values were converted from mmol/L to mg/dL (TG: ×88.57; FPG: ×18.00), but the original data is reported here as mmol/L for clinical relevance. WC was measured midway between the lowest rib and iliac crest at the end of a normal expiration using a non-stretchable tape, ensuring a snug fit without skin compression and parallel alignment to the floor. Two measurements were taken. If they differed by >1 cm, a third was obtained, and the average of the two closest values was recorded. neutrophil-to-lymphocyte ratio (NLR) was calculated as the absolute neutrophil count divided by the absolute lymphocyte count, both derived from complete blood count analysis.

### Variable definition

$\text{TyG index}=\text{Ln}\frac{\text{TG}(\text{mg}/\text{dl})\times \text{FPG}(\text{mg}/\text{dl})}{2}$ (15).

The diagnostic criteria for MASLD were based on the standards outlined in the “A Multisociety Delphi Consensus Statement on New Fatty Liver Disease Nomenclature” (16). The diagnostic criteria for carotid plaque were based on the standards outlined in the “Recommendations for the Assessment of Carotid Arterial Plaque by Ultrasound for the Characterization of Atherosclerosis and Evaluation of Cardiovascular Risk of the American Society of Echocardiography” (17).

### Statistical analysis

Categorical variables were reported as numbers (percentages) and compared using χ²-tests. The normality of continuous variables was assessed using the Kolmogorov–Smirnov test. Non-normally distributed data were reported as medians and interquartile range (IQR) and compared using the Mann–Whitney U-test. The linearity of the relationship between age and the log odds of carotid plaque was assessed using the Box-Tidwell test. No significant deviation from linearity was found, supporting their use as continuous variables in the regression models. Baseline characteristics across MASLD status strata were evaluated by multivariable logistic regression models to assess the association between the TyG index and carotid plaque. The diagnostic performance of the TyG index was evaluated by receiver operating characteristic (ROC) curve analysis and DeLong tests to compare differences in the area under the curve (AUC) that were observed in the MASLD status strata. the TyG index cutoffs were optimized using Youden’s index. The statistical analysis was performed with SPSS 26.0 and MedCalc 20.0. Statistical significance set at a two-tailed *P*-value of < 0.05.

## Results

### Baseline characteristics

The study cohort included 3, 414 participants with a mean age of 46.00 years, and 43.20% were men. The MASLD prevalence was 23.3% (n=796). The MASLD group had a significantly higher metabolic risk profile, including an elevated body mass index (BMI) of 26.74 kg/m^2^ vs. 23.31 kg/m^2^), SBP of 129 mmHg vs. 121.

mmHg), DBP of 80 mmHg vs. 73 mmHg), the TyG index (8.81 vs. 8.29), triglycerides (TG) of 1.61 mmol/L vs. 1.01 mmol/L), fasting blood glucose (FBG) of 5.17 mmol/L vs. 4.95 mmol/L), and LDL-C of 3.02 mmol/L vs. 2.76 mmol/L) (all *P* < 0.001). Participants with MASLD were also more likely to be men, to smoke, and to consume alcohol (all *P* < 0.001). Of the total 3, 414 participants, 726 individuals (21.27%) were identified with carotid plaque. The prevalence was significantly higher in the MASLD group (211 participants, 26.51%) compared to the non-MASLD group (515 participants, 19.67%) (*P* < 0.001). All detected plaques were subclinical and asymptomatic, as defined by ultrasonographic criteria (17), which primarily assess plaque presence and morphology for cardiovascular risk stratification rather than clinical symptoms of obstruction (**Table 1**). Subsequently, we evaluated the prevalence of carotid plaque across different age strata. As anticipated, plaque prevalence increased markedly with age. Notably, within each age stratum, the prevalence was consistently higher in participants with MASLD compared to those without. Specifically, among young adults (<45 years), plaque was present at 3.62% (47/1298) of the non-MASLD group versus 7.36% (22/299) of the MASLD group. This pattern was more pronounced in middle-aged (45–60 years) participants 26.15% (234/895) vs 30.54% (102/334). Interestingly, in the elderly (>60 years) subgroup, the difference in plaque prevalence between MASLD and non-MASLD participants was attenuated and became non-significant (55.06% vs 54.04%, χ^2^=0.49, *P*=0.824).”and was highest in the elderly (>60 years) subgroup 55.06% (234/425) vs 54.04% (87/161).

**Table 1:** Baseline characteristics stratified by MASLD status.

Characteristics	Total (n=3414)	MASLD (n=796)	Non-MASLD (n=2618)	χ^2^/U	*P*
Gender, n (%)				62.938	<0.001
Male	1475(43.20)	441(55.40)	1034(39.50)		
Female	1939(56.80)	355(44.60)	1584(60.50)		
Age	46.00(21.00)	49.00(20.75)	45.00(21.00)	-5.782	<0.001
BMI, M(IQR)	24.11(4.41)	26.74(3.72)	23.31(3.95)	-26.425	<0.001
Smoking, n (%)				17.819	<0.001
Yes	438(12.83)	137(17.21)	301(11.50)		
No	2976(87.17)	659(82.79)	2317(88.50)		
Drinking, n (%)				33.546	<0.001
Yes	877(25.69)	267(33.54)	610(23.30)		
No	2537(74.31)	529(66.46)	2008(76.70)		
Pulse, M(IQR)	78.00(15.00)	79.00(16.00)	78.00(15.00)	-1.202	0.229
SBP, M(IQR)	123.00(21.00)	129.00(20.00)	121.00(22.00)	-12.409	<0.001
DBP, M(IQR)	75.00(15.00)	80.00(15.00)	73.00(14.00)	-12.750	<0.001
WC, M(IQR)	86.00(16.00)	94.00(12.00)	83.00(14.00)	-22.743	<0.001
WBC, M(IQR)	5.73(1.82)	6.20(1.80)	5.58(1.75)	-12.129	<0.001
NEUT, M(IQR)	3.15(1.31)	3.45(1.33)	3.05(1.28)	-9.581	<0.001
LYMPH, M(IQR)	1.93(0.71)	2.12(0.74)	1.89(0.68)	-10.075	<0.001
RBC, M(IQR)	4.55(0.59)	4.72(0.63)	4.51(0.58)	-11.348	<0.001
HGB, M(IQR)	138.00(20.00)	144.00(21.00)	136.00(19.00)	-11.935	<0.001
PLT, M(IQR)	233.00(71.00)	239.00(73.75)	230.00(70.25)	-4.517	<0.001
TG, M(IQR)	1.12(0.72)	1.61(0.83)	1.01(0.58)	-24.024	<0.001
LDL, M(IQR)	2.82(1.04)	3.02(1.03)	2.76(1.02)	-7.551	<0.001
TC, M(IQR)	4.72(1.16)	4.88(1.19)	4.68(1.14)	-5.138	<0.001
HDL, M(IQR)	1.41(0.40)	1.29(0.32)	1.46(0.40)	-13.282	<0.001
FBG, M(IQR)	5.00(0.67)	5.17(0.69)	4.95(0.64)	-11.176	<0.001
TyG, M(IQR)	8.41(0.66)	8.81(0.53)	8.29(0.60)	-25.009	<0.001
Carotid Plaque, n (%)				17.037	<0.001
Yes	726(21.27)	211(26.51)	515(19.67)		
No	2688(78.73)	585(73.49)	2103(80.33)		

Data are medians (IQR) or n (%). *P*-values from Mann–Whitney U or χ² tests.

### Association between the TyG index and carotid plaque

The neutrophil-to-lymphocyte ratio (NLR) was included as a covariate given its established role as an inflammatory biomarker. Elevated NLR levels reflect systemic inflammation that directly promotes endothelial dysfunction and plaque vulnerability in atherosclerosis (18, 19). Notably, upon comprehensive adjustment for covariates including waist circumference, the association between the TyG index and carotid plaque in the entire cohort was attenuated and was no longer statistically significant (fully adjusted OR: 1.254, 95% CI: 0.985–1.596; *P*=0.066). However, and most importantly, stratification by MASLD status revealed a pronounced effect modification. In the non-MASLD group, the TyG index remained a strong and significant predictor of plaque (fully adjusted OR: 1.436, 95% CI: 1.066–1.935; *P*=0.017). In stark contrast, the TyG index was not significantly associated with plaque in the MASLD group (fully adjusted OR: 0.746, 95% CI: 0.453–1.228; *P*=0.249) (**Table 2**). Critically, the interaction term (TyG index × MASLD status) was significant in the fully adjusted model (OR: 0.532, 95% CI: 0.303–0.937; *P* =0.029), confirming that MASLD status is a key modifier that attenuates the association of the TyG index with carotid plaque (**Table 3**).

**Table 2:** Logistic regression for the TyG index and carotid plaque, stratified by MASLD status.

Groups	OR (95%CI)	*P*
Total population		
Model 1	2.416(2.008-2.907)	<0.001
Model 2	1.385(1.107-1.733)	0.004
Model 3	1.254(0.985-1.596)	0.066
Non-MASLD group		
Model 1	2.894(2.289-3.658)	<0.001
Model 2	1.537(1.155-2.045)	0.003
Model 3	1.436(1.066-1.935)	0.017
MASLD group		
Model 1	1.334(0.877-2.030)	0.178
Model 2	0.786(0.481-1.284)	0.336
Model 3	0.746(0.453-1.228)	0.249

Model 1: Crude

Model 2: Adjusted by age and sex

Model 3: Adjusted by age, sex, BMI, SBP, DBP, WC, smoking status, drinking status and NLR

**Table 3:** Multivariable logistic regression analysis of the TyG index, MASLD, and their interaction term for carotid plaque.

Characteristic	*β*	SE	Wald	OR (95%CI)	*P* value
TyG index	0.350	0.150	5.413	1.422 (1.049-1.928)	0.023
TyG X MASLD	-0.630	0.288	4.782	0.532 (0.303-0.937)	0.029
MASLD	0.095	0.126	0.566	1.099 (0.859-1.407)	0.452
Sex	0.352	0.155	5.141	1.422 (1.049-1.928)	0.023
BMI	-0.036	0.027	1.761	0.965 (0.915-1.017)	0.185
Age	0.090	0.005	354.045	1.094 (1.084-1.105)	<0.001
SBP	0.006	0.005	1.262	1.006 (0.996-1.016)	0.261
DBP	0.015	0.007	4.747	1.016 (1.002-1.030)	0.029
Smoking	0.225	0.142	2.514	1.252 (0.948-1.652)	0.113
Drinking	0.173	0.139	1.541	1.189 (0.905-1.561)	0.214
NLR	-0.047	0.068	0.482	0.954 (0.834-1.090)	0.488
WC	0.008	0.010	0.601	1.008(0.988-1.027)	0.438

After comprehensive adjustment for age, sex, BMI, blood pressure, smoking, drinking, and NLR, WC, the index–MASLD interaction remained significant (OR=0.532, 95%CI: 0.303-0.937; *P*=0.029). This confirms that the MASLD effect modification is independent of the metabolic and lifestyle confounders. Notably, age emerged as the strongest carotid plaque predictor (OR=1.095, *P* < 0.001), while DBP had a modest independent association (OR=1.015, *P*=0.030).

### Diagnostic performance of the TyG index

**Figure 2:**
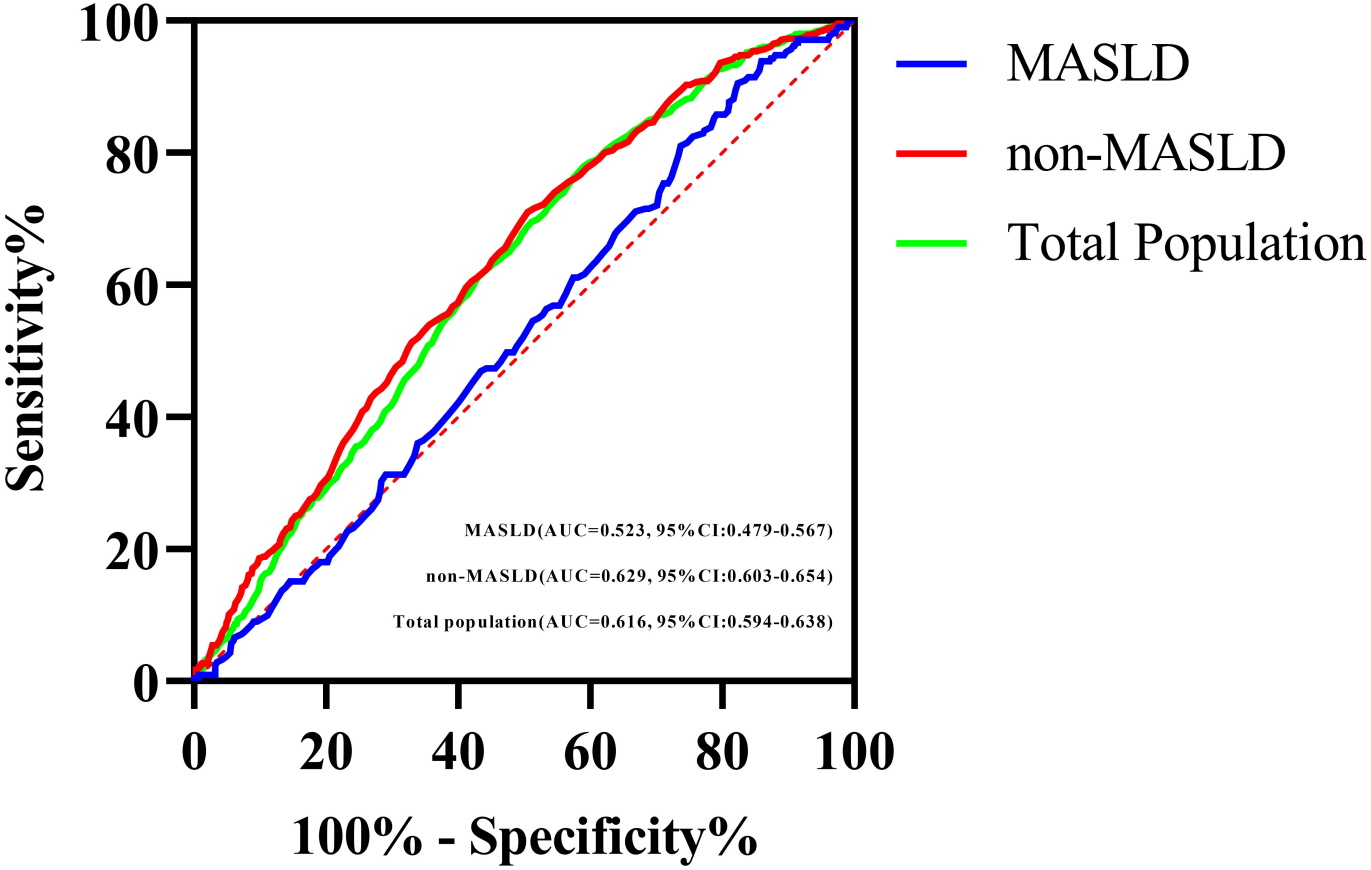
ROC curves of the TyG index for predicting carotid plaque in the total study population, the MASLD population, and the non-MASLD population.

The AUC for the prediction of carotid plaque by the TyG index was smaller in MASLD group (0.523, 95% CI: 0.479–0.567) than in the non-MASLD group (0.629, 95% CI: 0.603–0.654; *P* < 0.001, DeLong test) (**Table 4**; **Figure 2**). To further investigate the impact of the severity of underlying liver disease on the predictive performance of the TyG index, we performed a sensitivity analysis, stratifying the cohort by steatosis grade. This revealed that the observed decrease in the AUC within the MASLD group was directly correlated with increasing steatosis severity. Specifically, the AUC decreased progressively from subjects without steatosis (0.629), to those with mild steatosis (0.534), and further to those with moderate-severe steatosis (0.530; trend *P* < 0.001). Critically, the TyG index demonstrated no discriminative capacity for carotid plaque detection in MASLD patients (AUC=0.523, *P*=0.316), effectively performing no better than random chance. This null predictive performance starkly contrasts with its significant utility in non-MASLD individuals (AUC=0.629, *P* < 0.001).

**Table 4:** Diagnostic performance of the triglyceride–glucose index for carotid plaque across study groups.

Groups	AUC (95%CI)	Sensitivity	Specificity	Cutoff value	*P* value
Total Population	0.616 (0.594-0.638)	78.0%	41.0%	8.245	<0.001
MASLD	0.523 (0.479-0.567)	90.5%	82.4%	8.365	0.316
Non-MASLD	0.629 (0.603-0.654)	71.1%	49.5%	8.245	<0.001

## Discussion

This study demonstrates that MASLD significantly modifies the association between the TyG index for carotid plaque. While the TyG index remained significantly associated with carotid plaque in individuals without MASLD, its predictive value was nullified in those with MASLD, indicating a critical-effect modification by hepatic steatosis status. This observation suggests that MASLD may introduce distinct pathophysiological pathways in carotid plaque development that are not fully captured by the TyG index. However, the suggestion of pathways potentially independent of classical IR mechanisms must be tempered by the absence of direct insulin sensitivity measures (e.g., HOMA-IR, euglycemic clamp). It is possible that MASLD may decouple the TyG index from the true IR (i.e., the index becomes a less accurate surrogate) rather than activating entirely IR-independent pathways.

Our findings align with and significantly extend the current understanding of the TyG index as a cardiometabolic risk marker. Our results are consistent with those of an international study by Lopez-Jaramillo et al. (20) that confirmed the TyG index as a reliable surrogate for IR and a predictor of cardiovascular events in the general population. We confirmed that the TyG index was strongly associated with carotid plaque in the overall study population (adjusted OR 1.427, *P*=0.0018), which reinforces the potential utility of the TyG index as an accessible indicator of atherosclerosis risk in the general population (13, 14, 21).

However, our study reveals a key nuance that was not previously described, the differential performance of the TyG index based on MASLD status. A previous large, cross-sectional study by Lu et al. (22), reported the association of the TyG index with carotid atherosclerotic plaque without stratifying by MASLD status. Our analysis demonstrates that lack of stratification overlooks a critical modifying factor, which is the absence of a significant association in the MASLD group (adjusted OR 0.746, *P*=0.249) and the statistically significant TyG index x MASLD interaction term (OR 0.532, *P*=0.029). Our findings provide new evidence that MASLD modifies the relationship between the TyG index and vascular pathology. To further explore the specificity of this effect, we conducted a supplementary analysis using the TG/HDL-C ratio, a well-established marker of atherogenic dyslipidemia. In contrast to the TyG index, the TG/HDL-C ratio was not significantly associated with carotid plaque presence in either the overall cohort or when stratified by MASLD status (**Supplementary Table 1**). This divergence underscores a critical nuance: the modifying effect of MASLD appears to be specific to metabolic surrogates that incorporate glucose metabolism (such as the TyG index), rather than applying universally to all lipid-derived risk markers. It also suggests that in our study population, the pathway linking atherogenic dyslipidemia per se to carotid plaque may be less prominent than the pathway involving insulin resistance and glucose homeostasis, which is uniquely disrupted by the MASLD milieu.

An intriguing and novel finding of our study was the attenuation of the difference in carotid plaque prevalence between MASLD and non-MASLD groups among the eldest participants (aged >60 years). While MASLD was consistently associated with a higher plaque burden in young and middle-aged adults, this association was absent in the elderly subgroup, with prevalence rates converging at 54.04% vs. 55.06% (*P*=0.824).This observed effect may be substantially influenced by survivorship bias (23), a form of selection bias wherein a competing risk (e.g., premature cardiovascular mortality) selectively removes high-risk individuals from the population over time. It is well-established that individuals with MASLD face an elevated risk of early cardiovascular events and mortality (24). Consequently, those who survive into advanced age likely represent a “resilient” or “selected” subgroup possessing a more favorable cardiometabolic phenotype, which attenuates the observable atherosclerotic risk attributable to MASLD itself (25). Alternatively, the overwhelming effect of advanced vascular aging and the universal accumulation of age-related risk factors may eclipse the additional risk contribution conferred by MASLD in older adults. This finding underscores the complex and dynamic interplay between MASLD, aging, and cardiovascular disease, suggesting that the risk associated with MASLD is not static but evolves across the life course and is susceptible to modification by competing events and selective survival (23, 25).

The attenuation effect resonates conceptually with emerging insights into the effects of metabolic dysfunction in MASLD patients. MASLD is increasingly recognized as not only a hepatic condition but also a systemic metabolic disruptor (26, 27). Samuel and Shulman’s foundational work illustrates how hepatic steatosis impairs insulin signaling pathways (28) by potentially decoupling peripheral glucose and lipid metabolism, which are components captured by the TyG index, from their downstream vascular consequences (29, 30). Our results empirically support this paradigm of decoupling, suggesting that the metabolic milieu of MASLD uncouples the TyG index from its expected correlation with atherosclerosis.

The ROC analysis further supports this interpretation. The significantly lower AUC for the TyG index in predicting carotid plaque in the MASLD group (AUC 0.523) versus the non-MASLD group (AUC 0.629; DeLong *P* < 0.001) quantitatively demonstrates the reduced discriminatory ability. This finding parallels observations by Wong et al. (31), who confirmed that MASLD was an independent risk factor for cardiovascular disease, independent of other known risk factors such as IR and diabetes.

This study enhances the field through key methodological innovations, notably by rigorously assessing effect modification. We employed multivariable logistic regression, incorporating interaction terms to move beyond simple stratification and to formally test and confirm a significant statistical interaction between the TyG index and MASLD status. This robust approach, adjusted for major confounders including age, sex, BMI, blood pressure, smoking, alcohol consumption, and NLR, provides strong evidence of genuine biological effect modification (32). The overwhelming association of age with carotid plaque (OR=1.095, *P* < 0.001) aligns with vascular aging mechanisms. However, it did not attenuate the TyG index–MASLD interaction, underscoring the specificity of hepatic steatosis in disrupting the TyG index’s predictive biology. Diagnostic performance was comprehensively evaluated using ROC curve analysis, with statistically significant differences in AUC between strata rigorously compared using DeLong tests. Further pairwise comparisons across MASLD severity levels revealed a significant gradient, particularly between the non-MASLD and mild MASLD groups, which support a dose-response relationship. Importantly, the use of real-world health check-up data enhances external validity. Moreover, the application of standardized, widely accepted ultrasonographic criteria for defining both MASLD and carotid plaques ensures clinical applicability. Strict exclusion of individuals on lipid- or glucose-lowering medications and those with pre-existing cardiovascular disease, was implemented to minimize confounding, thereby strengthening the potential for causal inference.

The robustness of our core finding is evidenced by: (1) the consistency of the interaction effect across minimal and full adjustment models (OR=0.528, *P* =0.027); (2) a dose-response gradient in AUC attenuation with worsening steatosis severity (*P* < 0.001); and (3) the non-significance of the TyG index in MASLD despite adequate power (β-error < 10% for OR=0.783). Clinically, these findings necessitate refined cardiovascular risk stratification. Clinicians using the TyG index must account for MASLD status, as TyG is significantly associated with atherosclerosis only in individuals without MASLD. In contrast, the TyG index does not show a significant association with plaque in patients with MASLD. This highlights the need to incorporate MASLD as a key modifier in risk algorithms that employ the TyG index or similar metabolic indices (33). Furthermore, MASLD emerges as a systemic risk amplifier beyond the hepatic sequelae. Its “uncoupling” effect reveals a distinct metabolic phenotype wherein conventional markers behave atypically. This challenges the simplistic view of MASLD as a mere comorbidity and instead positioning it as a central modulator of metabolic-vascular crosstalk (30, 34). Consequently, therapeutic strategies solely targeting glucose or lipids (reflected by the TyG index) may be insufficient to address atherosclerosis risk in MASLD populations, prioritizing interventions that address underlying drivers (e.g., hepatic IR, inflammation, or lipoprotein remodeling) (35, 36). Theoretically, our results reinforce MASLD as a systemic disorder with a unique pathophysiology (14). This study provides human evidence that corroborates animal models demonstrating that hepatic steatosis alters systemic metabolic communication (28, 37). Overall, the results underscore the imperative for disease-specific risk models that integrate organ-specific metabolic dysfunction as effect modifiers.

Our study has several limitations. Its cross-sectional design precludes causal inferences regarding the TyG index, MASLD, and plaque progression. Future longitudinal studies are needed to clarify their temporal relationships. Although major confounders were adjusted for, residual confounding from unmeasured lifestyle and metabolic variables remains possible. The absence of direct insulin sensitivity measures also limited mechanistic exploration. Nonetheless, such factors are unlikely to explain the robust interaction between TyG index and MASLD status.The single-center, retrospective nature may affect generalizability, and external validation in diverse cohorts is warranted. The lack of significant AUC differences between MASLD severity groups may be due to limited sample size and the diagnostic constraints of ultrasound. Future studies should incorporate advanced imaging and biomarkers to improve severity stratification. Finally, excluding patients on lipid- or glucose-lowering medications enhances internal validity but may reduce clinical generalizability, as many MASLD patients require such treatments. Future research including medicated patients, is necessary to confirm our findings in real-world populations. Prospective studies combining multi-omics, advanced imaging, and precise insulin assays would help elucidate the mechanisms behind MASLD-related effect modification.

## Conclusion

In conclusion, this study demonstrates that MASLD acts as a significant effect modifier, substantially attenuating the association of the TyG index with carotid plaque. This suggests that future risk assessment models should account for the presence of MASLD when evaluating the TyG index. Future cardiovascular risk stratification algorithms should account for the presence of MASLD to avoid underestimating risk in individuals with MASLD or misinterpreting TyG index results in MASLD patients. Beyond refining prediction tools, our findings highlight the profound systemic metabolic disruption associated with MASLD, which may alter the relationship between established metabolic markers and their vascular consequences. Future research should prioritize longitudinal studies exploring the mechanistic basis of this uncoupling, particularly the roles of hepatic insulin resistance, altered lipid metabolism, and inflammation, and investigate novel biomarkers or imaging techniques that remain predictive within the distinct metabolic environment of MASLD. Future cardiovascular risk assessment in MASLD patients may need to rely more on alternative strategies, such as imaging biomarkers. Ultimately, integrating these insights into personalized risk assessment models holds promise for optimizing cardiovascular prevention strategies for the growing population affected by MASLD.

## Data Availability

The raw data supporting the conclusions of this article are not publicly available due to ethical reasons/patient privacy concerns as specified in the ethical approval and participant consent forms. However, de-identified data will be made available by the corresponding author upon reasonable request.
