# Supplementary material for: Metabolic dysfunction-associated steatotic liver disease attenuates the predictive value of the triglyceride–glucose index for carotid plaque: evidence of insulin resistance-independent pathways
Source: Front Endocrinol (Lausanne). 2025 Oct 16;16:1696652. doi: 10.3389/fendo.2025.1696652 (PMC12571656; doi:10.3389/fendo.2025.1696652)
Supplement: Supplementary file 1 [file Table1.docx]

**Supplement Table 1** Logistic regression for the TG/HDL-C index and carotid plaque, stratified by MASLD status

| Groups | OR (95%CI) | *P* |
| --- | --- | --- |
| **Total Population** |  |  |
| Model 1 | 0.978(0.857-1.117) | 0.745 |
| Model 2 | 0.924(0.791-1.080) | 0.322 |
| Model 3 | 0.923(0.790-1.079) | 0.315 |
| **Non-MASLD Group** |  |  |
| Model 1 | 0.988(0.846-1.155) | 0.882 |
| Model 2 | 0.903(0.749-1.088) | 0.284 |
| Model 3 | 0897(0.744-1.082) | 0.257 |
| **MASLD Group** |  |  |
| Model 1 | 0.945(0.734-1.218) | 0.664 |
| Model 2 | 0.970(0.731-1.288) | 0.835 |
| Model 3 | 0.978(0.734-1.305) | 0.881 |

Model 1: Crude

Model 2: Adjusted by age and sex

Model 3: Adjusted by age, sex, BMI, SBP, DBP, WC, smoking status, drinking status and NLR
